# A novel high-temperature furnace for combined *in situ* synchrotron X-ray diffraction and infrared thermal imaging to investigate the effects of thermal gradients upon the structure of ceramic materials

**DOI:** 10.1107/S1600577514014209

**Published:** 2014-08-15

**Authors:** James B. Robinson, Leon D. Brown, Rhodri Jervis, Oluwadamilola O. Taiwo, Jason Millichamp, Thomas J. Mason, Tobias P. Neville, David S. Eastwood, Christina Reinhard, Peter D. Lee, Daniel J. L. Brett, Paul R. Shearing

**Affiliations:** a Electrochemical Innovation Laboratory, Department of Chemical Engineering, UCL, London WC1E 7JE, UK; b Research Complex at Harwell (RCaH), Rutherford Appleton Laboratory, Didcot, Oxfordshire OX11 0FA, UK; c School of Materials, University of Manchester, Oxford Road, Manchester M13 9PL, UK; d Diamond Light Source, Harwell Science and Innovation Campus, Didcot, Oxfordshire OX11 0DE, UK

**Keywords:** X-ray diffraction, solid oxide fuel cell, infrared imaging, thermal imaging, stress analysis, composite materials

## Abstract

A new technique combining *in situ* X-ray diffraction using synchrotron radiation and infrared thermal imaging is reported. The technique enables the application, generation and measurement of significant thermal gradients, and furthermore allows the direct spatial correlation of thermal and crystallographic measurements. The design and implementation of a novel furnace enabling the simultaneous thermal and X-ray measurements is described. The technique is expected to have wide applicability in material science and engineering; here it has been applied to the study of solid oxide fuel cells at high temperature.

## Abstract 2

A combined X-ray diffraction and thermal imaging technique is described to investigate the effect of thermal gradients on high-temperature composite materials.

## 1. Introduction   

There is a general class of problem involving interfacial mechanical deformation in composite materials owing to coefficient of thermal expansion mis-match, which can generate internal stresses that are often sufficient to cause local internal damage or even complete fracture. These stresses cannot be measured by conventional techniques and therefore are poorly understood (Ishihara *et al.*, 2002 ▶). In order to improve the understanding of these composite structures, internal stress distributions and internal fracture processes need to be studied non-destructively and *in situ*. Synchrotron radiation provides a powerful toolset for the study of such materials due to the flexible nature of many beamlines which enable *in situ* measurements *via* integrated furnace designs; in addition, the high-resolution measurements available allow internal changes in lattice structure to be measured with high spatial and temporal accuracy.

One such device which utilizes a compound structure is the solid oxide fuel cell (SOFC). SOFCs are electrochemical energy conversion devices in which the half-reactions are supported by composite porous electrodes; these electrodes provide the triple phase boundaries between reactant gas phase, ionically conductive electrolyte and electrically connected catalyst necessary for the electrochemical reaction to proceed.

**Figure 1:**
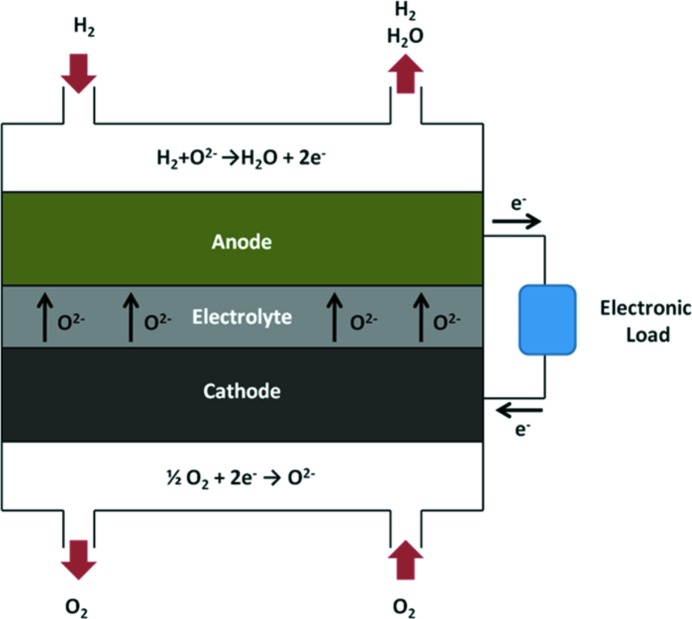
Schematic of SOFC operation illustrating the electrochemical half-cell reactions and associated transport of O^2−^ ions from the cathode through the dense electrolyte to the anode with the corresponding electron path through an external load highlighted.

SOFCs typically operate at temperatures in excess of 600°C in order for the electrode to become sufficiently ionically conductive. A schematic of SOFC operation is shown in Fig. 1 ▶ and the reader is referred to Shearing *et al.* (2010 ▶) for further background information.

In a working cell, the porous electrodes are deposited on either side of a dense ceramic membrane, typically yttria-stabilized zirconia (YSZ); the anode, which supports the hydrogen oxidation reaction, is typically a Ni–YSZ composite (cermet). As there is a substantial mis-match in thermal expansion coefficient between these components, operation at high temperatures and thermal cycling poses significant challenges. Mechanical failure of SOFC components has been attributed to a range of stresses between the component layers within cells (Clague *et al.*, 2012 ▶; Lin *et al.*, 2007 ▶; Liu *et al.*, 2010 ▶; Selimovic *et al.*, 2005 ▶; Villanova *et al.*, 2010 ▶). In addition to these, the presence of thermal gradients during the heating/cooling phases has also been reported as being a significant cause of failure in SOFCs (Apfel *et al.*, 2006 ▶; Nakajo *et al.*, 2009 ▶). These problems are not unique to SOFCs, and thermal gradients are known to promote failure in a range of cermet materials; for example, thermal barrier coatings (Qian *et al.*, 1998 ▶), which are used in a range of applications including gas turbines (Hutchison & Evans, 2002 ▶) and automotive components (Kokini *et al.*, 1997 ▶).

Synchrotron radiation allows *in situ* high angular and spatial resolution X-ray diffraction to be conducted to investigate the effect of thermal phenomena on SOFCs as a function of position within the sample; however, to date, reports in the literature of synchrotron radiation for this purpose are limited (Sumi *et al.*, 2005 ▶; Tanaka *et al.*, 2008 ▶; Villanova *et al.*, 2012 ▶).

Recently, measurement of crystallographic strain under thermal gradients has been achieved using another technique, the coupling of infrared lamp heat sources with measurements using thermocouple arrays (Li *et al.*, 2006 ▶; Siddiqui *et al.*, 2013 ▶).

This work represents the first time a combined X-ray diffraction and infrared imaging study has been performed. The combination of these techniques, utilizing a novel furnace design, enables high-resolution synchrotron data to be collected with the simultaneous recording of spatially resolved temperature data. In addition, the use of infrared imaging enables non-contact direct surface measurement; eliminating the necessity for thermocouple arrays, required to record spatially resolved thermal data (which may act as heat sinks), while also simplifying the design of the experiment.

This method enables the investigation of the effect of one-dimensional thermal gradients by sub-dividing the sample into a range of isotherms and analysing the effect of thermal gradients upon lattice parameters within a sample; in this case a composite Ni/YSZ SOFC electrode.

## 2. Materials and methods   

Throughout the experiments a ‘half-cell’ SOFC configuration was used in order to measure the effects of the anode/electrolyte interface in isolation of the cathode. Commercially available anode supported planar NiO/YSZ half cells (AEB-27; Fuel Cell Materials, Ohio, USA) were prepared by laser cutting into individual tokens of dimensions 10 mm × 10 mm (Laser Micromachining Ltd, Denbighshire, UK). The cell was constructed of a 6–10 µm layer of YSZ-8 electrolyte on a 220–260 µm NiO/YSZ anode layer. The cells were reduced at 600°C in 10% H_2_ in N_2_ for 3 h.

**Figure 2:**
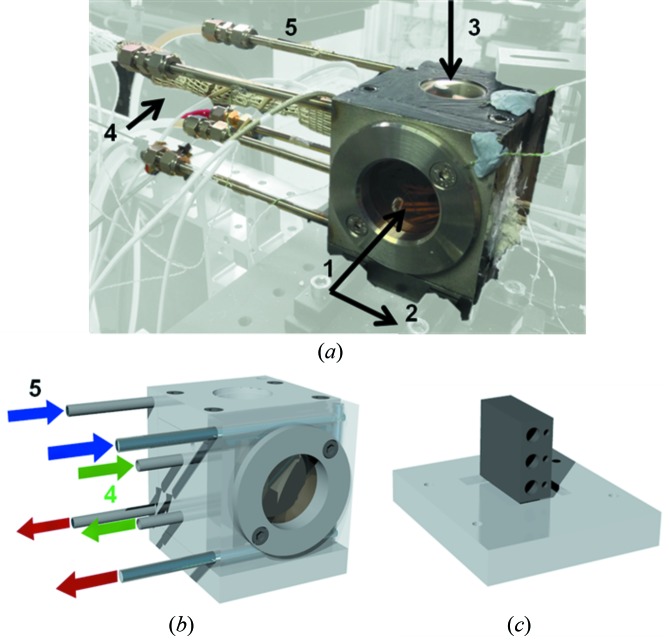
Photograph (*a*) and schematic design (*b*) of the furnace enclosure which is mounted over the internal furnace stage (*c*) highlighting: (1) the beam direction, *i.e.* direction of incident X-rays, and (2) the scan direction, *i.e.* the direction of stage translation in the vicinity of the beam-side Kapton window. The IR imaging direction is given by the arrow located at 3, with the external portion of the cooling circuit and gas preheating line located at 4 and 5, respectively. (*c*) The internal furnace stage with slots for three 225 W cartridge heaters.

In order to conduct the experiment at relevant SOFC operating temperatures, a novel furnace was designed which enabled concurrent infrared and X-ray imaging; the furnace is shown in Fig. 2 ▶. The samples were heated using an Inconel sample stage, which was heated by three PID-controlled 225 W cartridge heaters (CIR-10121/240V; Omega Engineering Inc., Connecticut, USA). The heating stage was placed upon a thin (*ca*. 5 mm) pyrophyllite insulating platform which sat within a recess located in the base of the furnace to aid with image alignment. The heaters were utilized in either a singular or parallel configuration in order to achieve sufficiently high, and stable, temperatures for the duration of each scan.

The furnace enclosure was composed of an Inconel shell which mounted the heating stage and bolted into position with the primary beamline rotation stage. The body contained two removable Kapton windows for X-ray transmission (arrow 1) and a recess for infrared imaging (arrow 3), into which a high-temperature IR transparent CaF_2_ window was placed (Z267589-1EA; Sigma Aldrich, St Louis, USA). Controlled motion was achieved using the primary beamline stage, enabling an accurate relationship to be inferred between the location of the thermal imaging camera (which was bolted to the same platform) and the relevant scan point.

In order to ensure the windows were not damaged by the high temperatures observed in the furnace, a cooling water loop was incorporated into the shell. The cooling system consisted of two 1/4" parallel streams of chilled water that passed through the shell of the furnace body and around the Kapton windows; the coolant was recycled through a chiller *via* Swagelok connections. In doing this, the temperatures of the Kapton windows, which were measured using K-type thermocouples, were kept below 100°C at all times ensuring the windows did not degrade or melt.

A forming gas mixture of 4% H_2_ in N_2_ was passed over the samples at all times at a rate of 80 ml min^−1^ to prevent oxidation of the Ni within the samples; trace amounts of NiO were measured from the diffraction pattern, and were found to be constant throughout the experiment. The forming gas was preheated to over 250°C using an inline heater in order to maintain a high ambient temperature within the furnace and consequently to reduce the duty of each cartridge heater. The furnace and gas lines were insulated to minimize heat losses.

Temperature control of the PID heaters was maintained using three K-type thermocouples located within 2 mm of the heaters in the block; a further four K-type thermocouples were located throughout the furnace to monitor the temperatures of the external surfaces.

Both the emissivity of the sample and transmittivity of the window were measured by calibrating the thermal imaging results with readings obtained from a thermocouple located on the surface of the sample. This was imperative due to the wide range of temperatures observed, and the potential for both emissivity and transmittivity changes of the sample and window, respectively. This calibration involved relating the temperature of both the control thermocouples and the sample thermocouple to the output of the camera. In all cases it was found that the emissivity and transmittivity of the sample and window were in excess of 0.9, leading to highly accurate thermographic results. In order to aid the exclusion of external sources of infrared, the surface of the furnace was painted in a matt black paint leading to the external steel having an emissivity in excess of 0.98, effectively creating a black-body surface.

**Figure 3:**
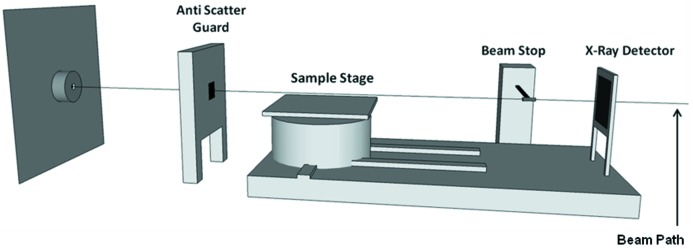
A schematic of the I-12 beamline highlighting the location of the major equipment within the experimental hutch. During the experiment the furnace was located on the sample stage allowing alignment with the beam.

X-ray diffraction (XRD) experiments were conducted at beamline I12 (JEEP) at the Diamond Light Source. During all experiments a monochromatic beam with an energy of 80 keV was used. A Thales detector (Pixium RF4343, Thales Electron Devices SA, France) was used to collect diffraction patterns throughout the experiment. The two-dimensional detector consists of a CsI sintillator on an amorphous Si substrate with an active area of 42.6 cm × 42.6 cm and a pixel size of 148 µm. The detector was positioned off-centre to the incident beam, enabling diffraction images to be captured over a 360° angle, with reciprocal space coverage up to a 2θ angle of 7.98°. A schematic of beamline I12 is shown in Fig. 3 ▶ illustrating the location of the major X-ray detection equipment, sample stage and furnace.

**Figure 4:**
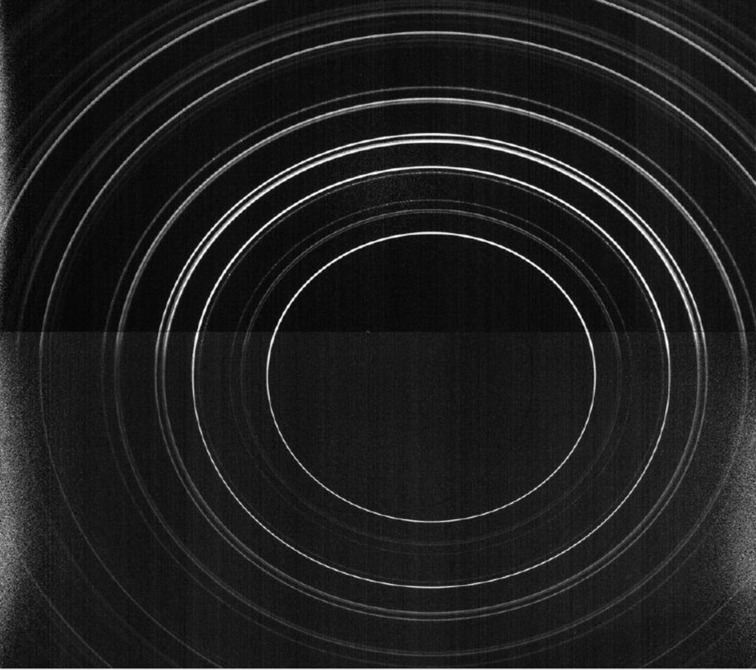
Two-dimensional diffraction data collected at the I-12 beamline at the mid-point of the sample. Shadowing can be observed in the third and fourth quadrants; analysis was performed using data obtained in the 90° and 180° directions, respectively.

Once the sample was placed within the furnace, coarse visual alignment was performed using X-ray radiography, and the sample position was registered with the thermal camera in order to relate the scan location and thermal imaging pixel locations. After a coarse alignment was achieved a second alignment was performed by X-ray diffraction. Upon alignment between sample and X-ray beam, calibration of the X-ray detection system was undertaken using a CeO_2_ standard at the position where the sample was to be located. X-ray diffraction patterns were recorded using a 50 µm beam aligned along the interface of the SOFC anode and electrolyte. The size of the beam was sufficiently large to ensure the interface was analysed at all times. Diffraction patterns were recorded with an exposure time of 80 s in order to ensure adequate count levels which resulted in a total scan time of approximately 30 min; a sample diffraction pattern is shown in Fig. 4 ▶.

In total, 20 exposures were collected during each scan, in 0.5 mm steps across the sample. Due to the location of the sample cell on the thick Inconel sample stage (Fig. 2 ▶), shadowing of the diffraction rings was observed in the third and fourth quadrants, respectively, as seen in Fig. 4 ▶. This resulted in a useable diffraction pattern from 0° to 180° which enabled measurement of the Ni lattice parameters for both the in-plane (90°) and out-of-plane (180°) directions.

Simultaneous thermal imaging was performed using a camera (FLIR SC5000MB; FLIR Systems France, Croissy-Beaubourg, France) which was calibrated for the temperatures in question using two ranges, 15–250°C and 250–1500°C. These calibration settings were in turn modified, to a suitable range of expected temperatures for the given experiment, in order to obtain accurate thermal results. The camera has an extended wavelength detector allowing detection of infrared light within the range 2.5–7 µm. A 27 mm (F/3) lens was used throughout the imaging process, with the images recorded using commercially available software (FLIR *ResearchIR*; FLIR Systems, France). The noise-equivalent temperature difference of the camera, a measure of the signal-to-noise ratio, was of the order of 19 mK in the calibration range. The optical set-up of the thermal camera resulted in a pixel resolution of 74 µm, resulting in a resolution of approximately seven resolvable thermal gradients per scan point. Images were recorded at 20 s intervals to minimize data storage requirements. Thermal images were analysed using commercially available software (*ResearchIR*; FLIR ATS, France) and gradients were averaged throughout the duration of each scan in order to account for any oscillations due to the PID control.

**Figure 5:**
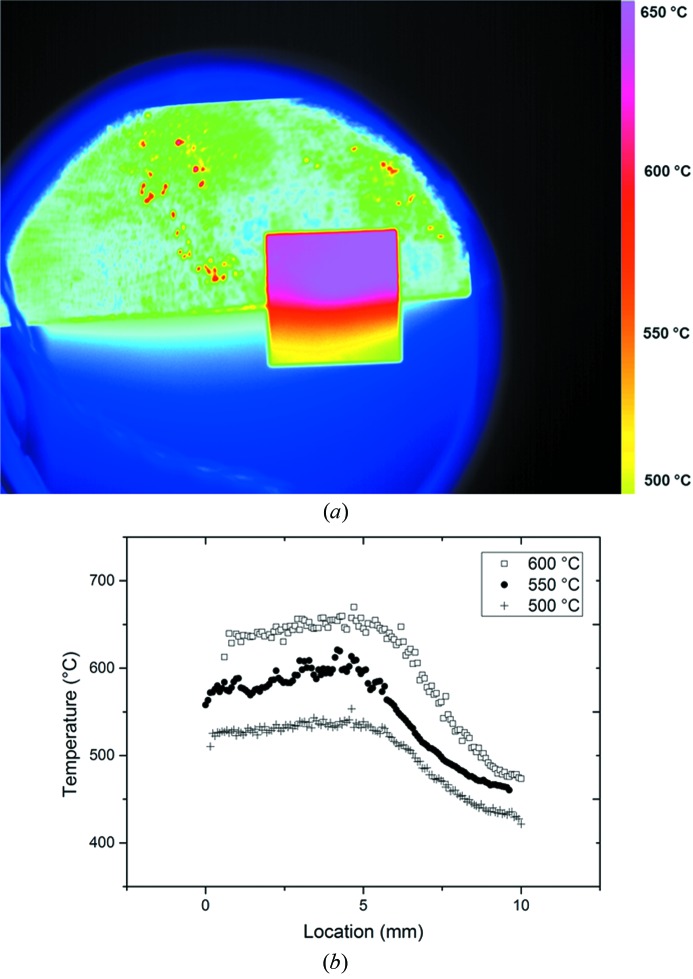
(*a*) Direction of the beam and scan shown with an example of a false-colour thermal image highlighting gradients using a 256 shade colour-map. (*b*) Thermal gradients obtained using infrared thermal imaging at three nominal scan temperatures as a function of location across the sample (from 0 to 10 mm) averaged along the isotherms and during the duration of the scan.

Thermal gradients along the specimen were achieved by overhanging the sample on the edge of the heating stage. Gradients were observed to be isothermal in the beam path with gradients observed along the scan path as indicated in Fig. 5 ▶. Nominal temperatures correspond to temperatures obtained with the furnace thermocouples; all other reported temperature values are obtained by means of IR imaging.

## 3. Results and discussion   

Using the experimental set-up described it was possible to simultaneously capture high-resolution XRD and thermal imaging data for samples with an imposed thermal gradient. The high spatial resolution of the thermal image enabled direct correlation of the beam position with sample temperature in a manner not previously possible with systems relying on bulk temperature measurements using thermocouples.

In addition to the significantly increased spatial resolution when compared with an array of thermocouples (*e.g.* Li *et al.*, 2006 ▶; Siddiqui *et al.*, 2013 ▶) the non-contact nature of the measurement enables a more accurate thermal reading as no heat sink is applied to the sample when measuring.

In total, diffraction data were collected at 20 discrete points along the scan path. Analysis of the crystallographic data was conducted using *Topas* (Bruker, Massachusetts, USA), to obtain the lattice parameter of the Ni face-centred cubic unit cell for both the in-plane (90°) and out-of-plane (180°) direction.

**Figure 6:**
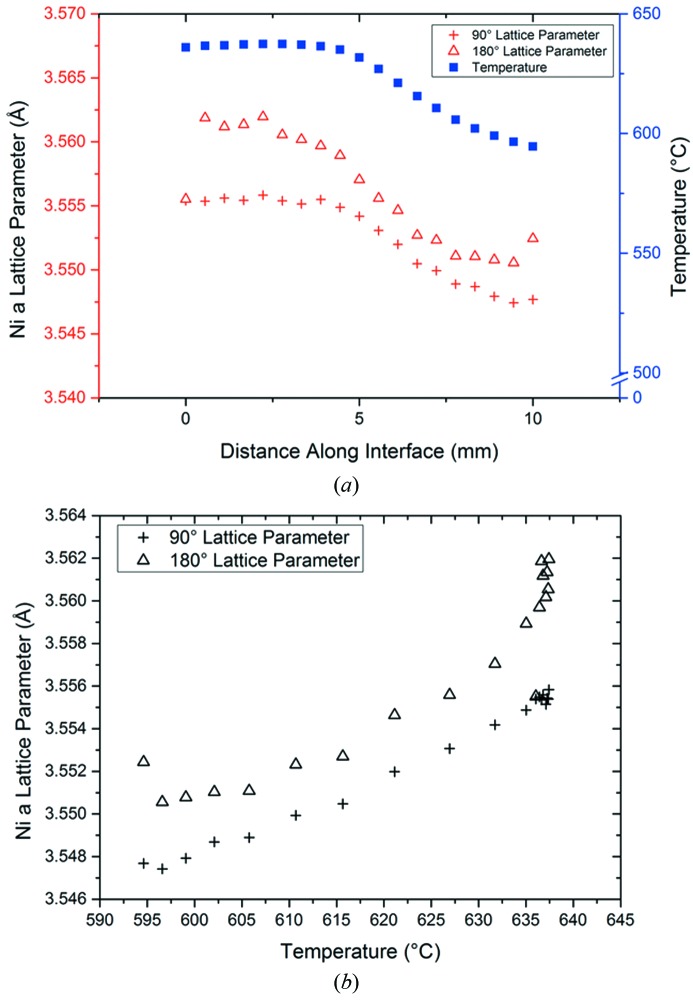
The effect of location along the sample from ‘hot’ to ‘cold’ (*a*) on both the in (180°) and out of (90°) plane stresses and thermal gradient at a PID controlled temperature of 640°C. Also shown is the Ni 90° and 180° lattice parameters as a function of temperature (*b*) at the same nominal temperatures as shown in (*a*).

Fig. 6 ▶ describes both the effect of spatial location (*a*) and temperature (*b*) on both the in-plane (90°) and out-of-plane (180°) Ni lattice parameters. At the 0 mm position, the specimen is in a uniform thermal condition without any thermal gradient. This position represents the ‘hottest’ area of the specimen. As the beam moves horizontally along the Ni/YSZ interface (increasing location value), the specimen is exposed to the thermal gradient. The onset of the thermal gradient (shown as blue squares) is seen to occur approximately half-way through the sample, with the final location (at 10 mm) being the ‘coldest’ area of the sample. Fig. 6(*b*) ▶ shows the same data plotted to indicate the temperature dependence of both Ni lattice parameters with temperatures obtained by means of IR imaging.

A decrease in both the 90° and 180° Ni lattice parameter corresponds with the decreasing temperature observed along the length of the sample (Fig. 6*a* ▶). This is in agreement with work performed previously (Suh *et al.*, 1988 ▶) which showed that the Ni lattice parameter decreases linearly with temperature. However, at the point co-incident with the highest thermal gradient (5–7 mm), convergence is observed between the 180° and 90° Ni lattice parameter (Fig. 6*b* ▶). The in-plane (90°) lattice parameter is observed to mirror the gradient, indicating a linear dependence of the lattice parameter on temperature as there is no thermal gradient in the 90° plane. In contrast, the 180° out-of plane lattice parameter displays non-linear behaviour, indicating contraction beyond what would be expected from purely thermal expansions. This is evident in Fig. 6 ▶ in the region 0–4 mm where the temperature is constant and the 180° lattice parameter decreases (but remains constant in the case of the isothermal 90° parameter).

This non-linear behaviour in the 180° plane is proposed to be as a result of a ‘relaying’ effect within the neighbouring unit cells in the sample. This increased contraction (in the 180° parameter) is due to the fact that the imposed thermal gradient is in the same plane. For a given unit cell, the contraction of the adjacent cell in the direction of increasing thermal gradient induces a reduction of lattice parameter beyond that expected due to pure thermal expansion of that cell. This effect is relayed across the whole sample such that there is still an additional contribution to the contraction of lattice parameter in the region (0–4 mm) where the temperature is constant. The observed non-uniformity in the in-plane and out-of plane strain responses will of course be exacerbated by the presence of the ceramic substrate which will constrain the sample. The technique described here is compatible with a comprehensive crystallographic strain mapping analysis; however, this is beyond the scope of the current work.

## 4. Conclusions   

A novel methodology is presented featuring a high-temperature furnace design for combined *in situ* X-ray diffraction and infrared thermal imaging enabling spatially resolved thermal and crystallographic measurements at high resolution. This technique presents significant benefits over the use of thermo­couples, enabling direct spatial correlation of the diffraction results and measured thermal data. Correlation of these two techniques allows the effect of both high temperatures and thermal gradients upon the lattice parameters to be investigated.

Here, the effect of high-temperature thermal gradients on solid oxide fuel cell anodes has been investigated; however, the technique can be applied to a wide range of samples and applications across different research areas involving composite materials.
